# Transgenic Mice Convert Carbohydrates to Essential Fatty Acids

**DOI:** 10.1371/journal.pone.0097637

**Published:** 2014-05-16

**Authors:** Victor J. Pai, Bin Wang, Xiangyong Li, Lin Wu, Jing X. Kang

**Affiliations:** 1 Laboratory for Lipid Medicine and Technology (LLMT), Massachusetts General Hospital and Harvard Medical School, Boston, Massachusetts, United States of America; 2 Cutaneous Biology Research Center, Massachusetts General Hospital and Harvard Medical School, Boston, Massachusetts, United States of America; CRCHUM-Montreal Diabetes Research Center, Canada

## Abstract

Transgenic mice (named “Omega mice”) were engineered to carry both optimized *fat-1* and *fat-2* genes from the roundworm *Caenorhabditis elegans* and are capable of producing essential omega-6 and omega-3 fatty acids from saturated fats or carbohydrates. When maintained on a high-saturated fat diet lacking essential fatty acids or a high-carbohydrate, no-fat diet, the Omega mice exhibit high tissue levels of both omega-6 and omega-3 fatty acids, with a ratio of ∼1∶1. This study thus presents an innovative technology for the production of both omega-6 and omega-3 essential fatty acids, as well as a new animal model for understanding the true impact of fat on human health.

## Introduction

There are several classes of fats that differentially affect human health, such as saturated fatty acids (SFA), monounsaturated fatty acids (MUFA), and polyunsaturated fatty acids (PUFA), including both essential omega-6 (n-6) and omega-3 (n-3) PUFA. Recent research has shown that our health is impacted not just by the quantity of fat consumed, but more significantly by the types of fat consumed [1],[2]. For example, the worldwide trend of increased SFA and n-6 PUFA intake and decreased n-3 PUFA intake has coincided with the growing prevalence of chronic diseases, such as heart disease, cancer, Alzheimer's, and diabetes [2].

Normally, mammals readily obtain SFA from either the diet or endogenous synthesis from glucose or amino acids [3], and MUFA can also be obtained from the diet or converted from SFA by the stearoyl-CoA desaturase-1 (SCD-1) gene [4]. On the other hand, n-6 and n-3 PUFA cannot be inter-converted or synthesized *de novo* in mammals and are mainly acquired through the diet [5]. The primary essential fatty acids in the diet are n-6 linoleic acid (18:2n-6, LA) and n-3 α-linolenic acid (18:3n-3, ALA), which can be converted into longer-chain n-6 PUFA arachidonic acid (20:4n-6, AA) and n-3 PUFA eicosapentaenoic acid (20:5n-3, EPA) and docosahexaenoic acid (22:6n-3, DHA), respectively, through a series of desaturation and chain-elongation enzyme systems [5]. The metabolites derived from n-6 and n-3 PUFA, namely eicosanoids, are functionally distinct and have important opposing physiological effects [6]. For example, n-6-derived eicosanoids generally promote inflammation, while n-3-derived eicosanoids have anti-inflammatory properties. Since the synthesis of n-6 and n-3 long-chain PUFA and their metabolites compete for the same enzymes, their ratio in body tissues determines the eicosanoid profile. Recent studies have indicated that the tissue n-6/n-3 PUFA ratio plays an important role in the pathogenesis of many chronic diseases [2],[7]. Many global health organizations now recommend an increased daily intake of n-3 PUFA, particularly EPA and DHA, but these n-3 PUFA are largely limited to marine sources (such as fish and algae) that can be less affordable or accessible [8]. Thus, it is often challenging to balance the different types of dietary fat in a way that optimizes health.

Unlike mammals, the roundworm *C. elegans* is capable of producing all classes of fatty acids as they possess the required genes. For example, the *fat-2* gene encodes a desaturase that catalyzes the conversion of MUFA to n-6 PUFA, and the *fat-1* gene enables the conversion of n-6 to n-3 PUFA [9]. Our laboratory previously created the *fat-1* transgenic technology to express the *fat-1* gene in mammals (both mice and livestock), which successfully demonstrated the conversion of dietary n-6 to n-3 PUFA and exhibited a balanced n-6/n-3 PUFA ratio in body tissues, regardless of diet [10],[11]. Our earlier work generated a unique *fat-1* mouse model for n-3 PUFA research and also provided a sustainable, land-based strategy for the production of n-3 PUFA from n-6 PUFA [12],[13],[14].

**Figure 1:**
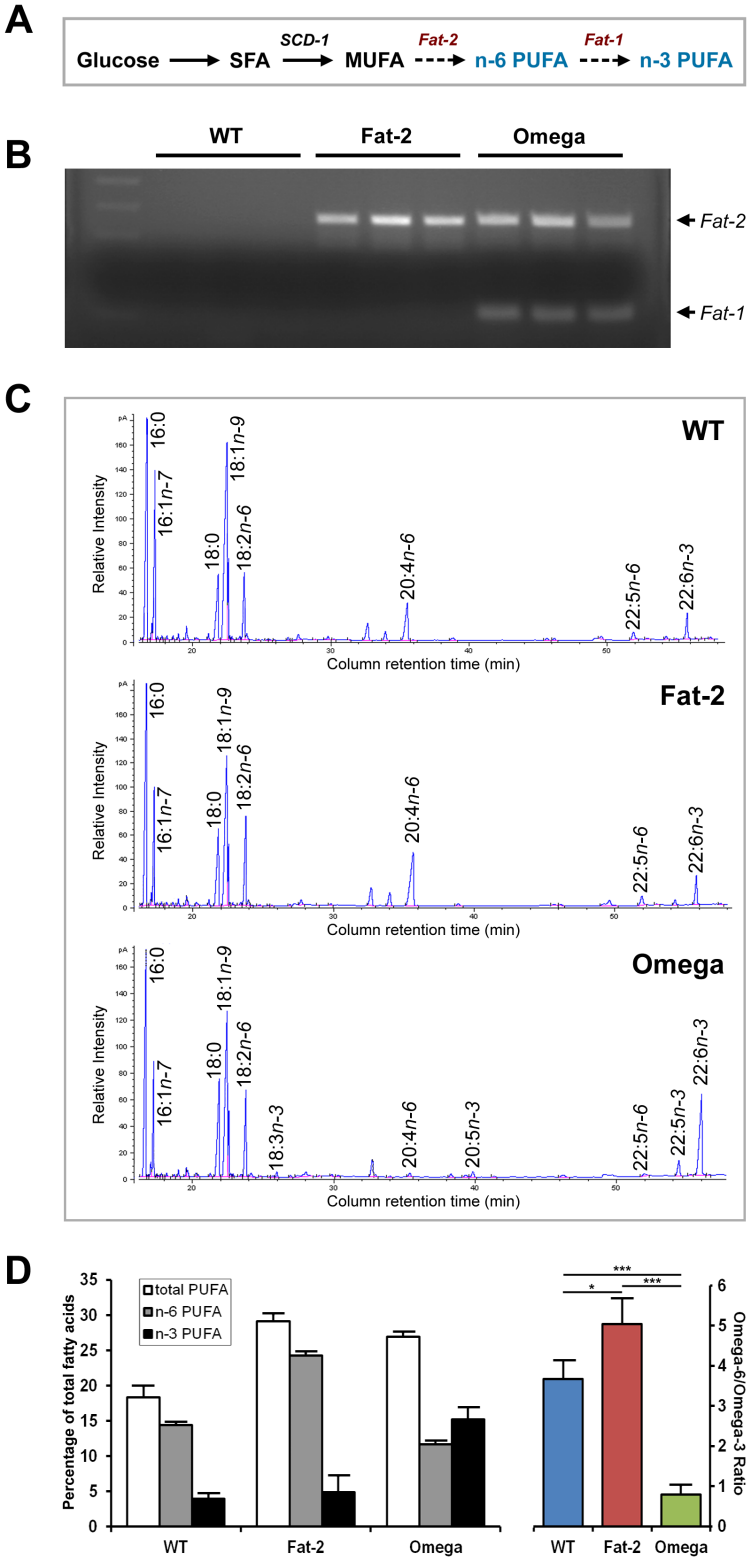
Generation, genotyping, and phenotyping of the *fat-2* and Omega transgenic mice. (**A**) Roadmap for the conversion of essential fatty acids from non-essential nutrients in Omega mice. In mammals, carbohydrates can be converted to SFA, and SFA can be converted into MUFA by SCD-1. Introduction of the *fat-2* and *fat-1* transgenes allows mammals to further convert MUFA into n-6 PUFA, and n-6 PUFA into n-3 PUFA, respectively. (**B**) Validation of *fat-1* and *fat-2* transgene expression in wild-type (WT), *fat-2*, and Omega mouse littermates by PCR. (**C**) Partial gas chromatograph traces showing the fatty acid profiles of total lipids extracted from skeletal muscles of a wild-type mouse (WT, upper panel), a *fat-2* transgenic mouse (*Fat-2*, middle panel), and an Omega transgenic mouse (Omega, lower panel). All mice were 10-week-old males and fed with the same diet high in SFA and carbohydrates and low in n-6 PUFA. (**D**) Quantification of PUFA from muscle tissue of WT, *fat-2*, and Omega mice (left). For significance values, refer to **Table 1** and **Table S2**. Comparison of the n-6/n-3 PUFA ratio among the phenotypes (right). Values expressed as mean ± s.d. (n = 3 per group; *P<0.05, **P<0.01).

The objective of this study was to generate a novel transgenic mouse model that can endogenously synthesize all essential fatty acids. Our strategy was to first create a *fat-2* transgenic mouse, possessing the *C. elegans fat-2* gene encoding an enzyme that converts MUFA into n-6 LA [9], and then cross the *fat-2* transgenic mice with *fat-1* transgenic mice, which we generated previously to possess the *C. elegans fat-1* gene, encoding an enzyme that converts n-6 to n-3 PUFA [10]. Through this procedure, we can generate a compound *fat-1*/*fat-2* transgenic mouse – hereafter referred to as the “Omega” mouse – that is capable of producing both n-6 and n-3 PUFA from a diet containing only saturated fat or carbohydrates (**Figure 1A**).

## Results and Discussion

To enable the higher expression of the *C. elegans fat-2* gene in mammals, we first optimized the codon of the *fat-2* sequence based on the mammalian desaturase sequence (**Figure S1**) and utilized a chicken beta-actin promoter to build a transgene construct (**Figure S2**), and then used microinjection to create the *fat-2* transgenic mouse. After the *fat-2* phenotype and genotype were confirmed, the heterozygous *fat-2* transgenic mice were backcrossed with C57BL6 wild-type (WT) mice for 5 generations, and then crossbred with heterozygous *fat-1* transgenic mice. The resulting offspring consisted of WT, *fat-1*, *fat-2*, and Omega mice. The *fat-1* mouse phenotype has been reported previously [7],[10],[12]. Expression of the *fat-1* and *fat-2* transgenes was validated by PCR, confirming that WT mice do not express any of the transgenes, the *fat-2* mice carry only the *fat-2* transgene, and the Omega mice express both the *fat-1* and *fat-2* transgenes (**Figure 1B**).

When WT, *fat-1, fat-2*, and Omega mouse littermates were fed the same diet high in saturated fat and carbohydrates and low in n-6 PUFA (**Table S1**), phenotype validation by gas chromatography (GC) revealed four distinct tissue fatty acid profiles (**Tables 1** **and** **2**). The difference in fatty acid profiles for the new *fat-2* and Omega mice are highlighted in **Figure 1C and D**. As expected, the WT mice exhibited high levels of saturated fat and very low levels of essential fatty acids, primarily n-6 PUFA, with an n-6/n-3 PUFA ratio of roughly 3.5. The *fat-2* transgenic mice displayed a significant increase in total tissue PUFA content, with the n-6 PUFA content in the muscle tissue doubling from about 700 µg/g to 1350 µg/g due to the conversion of oleic acid into n-6 LA and AA, without much change in n-3 PUFA levels, resulting in an n-6/n-3 PUFA ratio of about 5. The Omega transgenic mice also exhibited significantly increased total PUFA content, but the tissue content of n-3 PUFA was increased by roughly five-fold compared to their WT and *fat-2* littermates, due to the conversion of almost half the n-6 PUFA content into n-3 PUFA, with a markedly decreased n-6/n-3 PUFA ratio of 0.75 (**Figure 1D**). MUFA levels were accordingly reduced in the *fat-2* and Omega mice, showing that n-6 PUFA had been converted from MUFA. No significant differences in SFA levels were observed among the phenotypes. Detailed fatty acid profiles for the four genotypes (WT, *fat-1*, *fat-2*, and Omega) are shown in **Tables S2–5**. Furthermore, when fed a high-carbohydrate, no-fat diet (**Table S1**), the *fat-2* and Omega mice still exhibited significant tissue levels of n-6 and n-3 PUFA, respectively, confirming their ability to produce essential fatty acids when lacking dietary fat and given only carbohydrates (**Table 2**). The tissue abundance of n-6 PUFA in the *fat-2* mouse and of both n-3 and n-6 PUFA in the Omega mouse, despite the diet containing little of these fatty acids, thus demonstrates the capability of our transgenic mice to produce essential fatty acids from non-essential nutrients – MUFA, SFA, and even carbohydrates.

**Table 1:** Comparison of the fatty acid profiles of various tissues among the four genotypes.

Muscle						
	SFA	MUFA	Total PUFA	n-6 PUFA	n-3 PUFA	n-6/n-3
**WT**	38.96±0.98	42.87±1.99^##△△^	18.19±1.29^##△△^	14.23±1.59^## 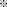 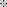 ^	3.96±0.48^△△ 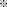 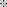 ^	3.60±0.95^△△ 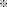 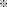 ^
**Fat-1**	40.51±0.35	42.28±1.81^▴▴^**	16.89±1.55^▴▴^**	6.23±1.16^ 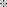 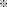 ▴▴^**	10.66±0.76^ 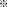 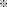 ▴▴^**	0.59±0.10^ 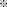 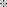 ▴▴^
**Fat-2**	41.15±2.02	29.71±2.13^##▴▴^	29.01±0.99^##★▴▴^	24.29±0.93^##★★▴▴^	4.72±0.40^★★▴▴^	5.18±0.52^★★▴▴^
**Omega**	41.08±0.47	33.03±2.12^△△^**	25.90±1.95^△△★^**	11.23±2.43^★★^**	14.67±2.09^△△★★^**	0.78±0.24^△△★★^

The four genotypes of mice were fed the same low-PUFA diet for about two months and tissue samples were subject to lipid analysis by gas chromatography. WT: Wild-type; SFA: saturated fatty acids; MUFA: monounsaturated fatty acids; PUFA: polyunsaturated fatty acids; n-6: omega-6; n-3: omega-3; n = 3 for each group;

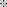
(WT vs Fat-1),

# (WT vs Fat-2),

△ (WT vs Omega),

▴ (Fat-1 vs Fat-2),

*(Fat-1 vs Omega),

★ (Fat-2 vs Omega), One symbol  = P<0.05, Two symbols  = P<0.01.

**Table 2:** Fatty acid profile of tail tissue from mice fed with a high-carbohydrate, no-fat diet.

	SFA	MUFA	Total PUFA	n-6 PUFA	n-3 PUFA	n-6/n-3
**WT**	28.85±7.64	64.09±8.13	7.06±0.89^##△△^	6.4±0.75^ 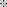 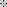 ##△△^	0.66±0.14^△△ 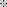 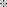 ^	9.93±1.2^##△△ 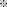 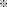 ^
**Fat-1**	28.24±0.33	65.46±1.05▴ *	6.32±0.71^▴▴^**	2.32±0.38^ 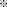 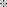 ▴▴^**	4±0.66^ 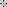 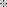 ▴▴^**	0.59±0.16^ 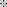 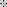 ▴▴^
**Fat-2**	27.78±1.56	57.14±2.01▴	15.1±0.65^##▴▴^	14.44±0.74^##★★▴▴^	0.65±0.09^★★▴▴^	22.44±3.82^##★★▴▴^
**Omega**	29.06±3.43	56.98±2.65*	13.97±0.78^△△^**	8.82±0.75^△△★★^**	5.16±0.28^△△★★^**	1.71±0.18^△△★★^

The four genotypes of mice were fed the same no-fat diet for about two months and tail tissue was subject to lipid analysis by gas chromatography. WT: Wild-type; SFA: saturated fatty acids; MUFA: monounsaturated fatty acids; PUFA: polyunsaturated fatty acids; n-6: omega-6; n-3: omega-3; n = 3 for each group;

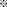
(WT vs Fat-1),

# (WT vs Fat-2),

△ (WT vs Omega),

▴ (Fat-1 vs Fat-2),

*(Fat-1 vs Omega),

★ (Fat-2 vs Omega), One symbol  = P<0.05, Two symbols  = P<0.01.

The Western human diet today is fundamentally different than it was throughout the majority of human evolution. Among the many shifts in dietary nutrients that have occurred over the last few decades, key changes include increases in saturated fat, carbohydrates, and n-6 PUFA intake with a decrease in n-3 PUFA intake [15],[16],[17]. As a result, modern humans have an n-6 to n-3 PUFA ratio that favors n-6 PUFA by as much as 20∶1; evolutionarily, this ratio would have been closer to 1∶1, and the discrepancy is thought to have profound physiological consequences [15],[16]. This study presents a new transgenic technology that could produce essential fatty acids, especially the beneficial n-3 PUFA, in animal products (such as meat, milk, eggs, etc.) by feeding animals with just carbohydrates and/or saturated fat. Given that n-3 PUFA are largely limited to marine sources (such as fish and algae), this technology could therefore generate sustainable and accessible n-3 PUFA resources, especially where only carbohydrates are available.

The transgenic technology presented in this study will also be of great utility in elucidating the impact of essential fatty acids on health. Conventionally, dietary modification is used to investigate the effects of different fatty acid profiles, which requires feeding animals with different diets. However, this method is problematic since the diets may not only contain different fatty acid compositions, but also variations in impurities, flavor, calories, or other components used between study groups, ultimately leading to confounding factors that complicate interpretation of results. Our transgenic technology allows us to create four different tissue fatty acid profiles by using a single diet, which makes it possible to evaluate the true health effects of different fats without the confounding factors of diet. This is very important for identification of metabolic biomarkers related to lipid metabolism.

This study clearly demonstrates the feasibility of producing mammals with the capability to convert SFA and MUFA to the essential n-6 and n-3 PUFA. The transgenic mice created by this project will serve as unique animal models, free of dietary confounding factors, for the reliable study of the biological effects of different fatty acid profiles. Ultimately, this transgenic technology serves as a new biotechnology for the production of essential fatty acids, especially n-3 EPA and DHA, to meet increasing demand.

## Materials and Methods

### Ethics statement

All animal procedures in this study were reviewed and approved by the Massachusetts General Hospital (MGH) Subcommittee on Research Animal Care (SRAC).

### Codon optimization

In order to efficiently express a gene from the lower-life C. elegans in mammals *in vivo*, the codon usage by *C. elegans* must be adjusted to match those used by mammals. The *fat-2* gene sequence was obtained from the gene bank (GenBank accession number NM_070159). We used mammalian desaturases as well as our previously optimized *fat-1* gene as references to determine the differences in codon usages of desaturases between C. elegans and mammals. We then manually adjusted the codon to achieve over 80% optimization (**Figure S1**).

### Gene synthesis

After modifying the gene sequence to optimize the codon usage, the *fat-2* gene was synthesized by GenScript (Piscataway, NJ). The gene was synthesized to be flanked with EcoR I digestion sites and delivered on pUC57 plasmid. After amplification, the *fat-2* sequence was inserted into a pCAGGS expression plasmid containing the chicken beta-actin promoter and cytomegalovirus enhancer (kindly provided by Dr. J Miyazaki, Osaka University Medical School). After ligation, the orientation was confirmed and the plasmid was amplified. Finally, the fragment containing the promoter, the *fat-2* sequence, and the polyA sequence was excised with SspI and Sfi I for microinjection (**Figure S2**).

### Microinjection

Transgenic mouse lines were produced at the MGH Transgenic Core Facility by injecting the purified *Ssp* I and *Sfi* I fragment into fertilized eggs from C57BL/6 X C3H mice. The fertilized eggs containing the transgene were transferred to pseudo-pregnant mice (B6CF1) to produce transgenic mice. The founder transgenic mice were then subjected to genotyping and phenotyping.

### Genotyping and phenotyping

Genotyping was carried out by removing the tip of the tail to acquire a DNA sample for RT-PCR, which was performed with the following primers: *fat-2* forward, GCGGCCA GACCCAGACCATC; and *fat-2* reverse, GGGCGAC GTGACCGTTGGTA. PCR products were run through gel electrophoresis on 2% agarose gel. Phenotyping by fatty acid composition analysis using gas chromatography (GC) was performed as previously described [18]. The *fat-2* mice were maintained after weaning on a low-PUFA diet (**Table S1**). GC was carried out after 20 days on the diet to allow for clearer phenotypes. Tissue samples were ground to powder under liquid nitrogen and total lipids were extracted using chloroform/methanol (2∶1, v/v). Fatty acids were then methylated by heating them at 100°C for 1 hour under 14% boron trifluoride (BF3)-methanol reagent (Sigma-Aldrich, St. Louis, MO) and hexane (Sigma-Aldrich). Fatty acid methyl esters were analyzed by GC using a fully automated 6890N Network GC System (Agilent Technologies, Santa Clara, CA) equipped with a flame-ionization detector and an Omegawax 250 capillary column (30 m×0.25 mm ID). Fatty acid standards (Nu-chek-Prep, Elysian, MN) were used to identify peaks of resolved fatty acids, and area percentages for all resolved peaks were analyzed using GC ChemStation Software (Agilent). The fatty acid C23:0 (20 µg/sample) was used as an internal standard to calculate the amount of each fatty acid measured.

After identifying the genotype and phenotype, the *fat-2* mice were mated with WT C57BL6 mice to create the F1 generation. The F1 generation was then backcrossed with WT C57BL6 mice at least 5 times in order to verify that the gene is transmittable as well as to establish a pure background, so that *fat-2* lines could be maintained with a significant phenotype. Each generation was subjected to genotyping by RT-PCR and phenotyping by GC.

### Generation of Omega mice

The compound *fat-1*/*fat-2* transgenic mice were created by crossbreeding heterozygous *fat-2* transgenic mice with heterozygous *fat-1* transgenic mice, which were previously generated by our group [10]. After weaning, the offspring were maintained either on low-PUFA diet or a no-fat diet (**Table S1**), and then genotyped by RT-PCR and phenotyped by GC. Genotyping by RT-PCR of the Omega mice was carried out with the following primers: *fat-1* forward, TGTTCATGCCTTCT TCTTTTTCC; *fat-1* reverse, GCGACCATACC TCAAACTTGGA; *fat-2* forward, GCGGCCA GACCCAGACCATC; *fat-2* reverse, GGGCGAC GTGACCGTTGGTA. Phenotyping by fatty acid composition analysis using GC was performed as previously described [18].

### Statistical analysis

GraphPad Prism 5 (GraphPad Software, San Diego, CA) was used for all statistical analyses. Data sets were analyzed by F-test to verify normal distribution. One-way ANOVA followed by the Tukey test was used to determine statistical significance, set at *P<0.05 and **P<0.01.

## Supporting Information

Figure S1 Optimized fat-2 sequence.
(DOC)

Figure S2 The pCAGGS plasmid and fat-2 construct for microinjection.
(DOC)

Table S1 Composition of the low-PUFA diet and the no-fat diet.
(DOC)

Table S2 Comparison of the muscle fatty acid profile of mice fed with a low-PUFA diet among the four genotypes.
(DOC)

Table S3 Comparison of the liver fatty acid profile of mice fed with a low-PUFA diet among the four genotypes.
(DOC)

Table S4 Comparison of the tail fatty acid profile of mice fed with a low-PUFA diet among the four genotypes.
(DOC)

Table S5 Comparison of the tail fatty acid profile of mice fed with a non-fat diet among the four genotypes.
(DOC)
