# Supplementary material for: Transgenic Mice Convert Carbohydrates to Essential Fatty Acids
Source: PLoS One. 2014 May 16;9(5):e97637. doi: 10.1371/journal.pone.0097637 (PMC4023978; doi:10.1371/journal.pone.0097637)
Supplement: Figure S1 — Optimized fat-2 sequence. (DOC) [file pone.0097637.s001.doc]

TAAAACTTGGCCCCCGACGAAGATGACAATCGCCACAAAAGTGAACACAAATAAAAAAGAC

CTGGATACGATCAAGGTGCCGGAGCTGCCAAGCGTGGCAGCTGTCAAAGCCGCAATCCCT

GAGCACTGCTTTGTGAAGGATCCCTTGACTAGCATTTCATATCTGATCAAGGATTACGTG

CTTCTCGCCGGTCTCTACTTTGCAGTTCCCTACATAGAGCATTATCTCGGATGGATCGGG

CTGCTTGGCTGGTATTGGGCCATGGGAATTGTTGGCTCCGCATTGTTCTGTGTGGGGCAC

GACTGCGGACACGGATCATTCTCCGATTATGAATGGCTCAATGATCTGTGTGGCCATTTA

GCCCATGCTCCTATTCTGGCTCCGTTCTGGCCCTGGCAGAAATCTCACCGCCAGCATCAC

CAGTACACATCCCACGTGGAAAAGGATAAGGGACACCCCTGGGTTACTGAGGAAGACTAC

AATAATCGCACCGCCATTGAGAAATATTTCGCCGTGATTCCAATTAGCGGATGGCTGCGA

TGGAATCCCATCTACACCATCGTCGGTCTGCCCGATGGCTCTCACTTCTGGCCTTGGTCC

CGGCTCTTCGAGACTACCGAGGATCGTGTCAAGTGTGCAGTTTCTGGCGTTGCATGCGCT

ATCTGCGCTTACATTGCCTTTGTGCTCTGCGACTATTCTGTCTACACATTTGTCAAGTAC

TACTACATTCCACTGCTCTTCCAGGGCCTGATCCTCGTCATTATCACGTATCTTCAACAC

CAGAATGAGGATATTGAGGTCTACGAAGCCGATGAGTGGGGCTTTGTACGCGGCCAGACC

CAGACCATCGACAGGCACTGGGGCTTCGGACTAGACAACATCATGCACAACATTACCAAC

GGTCACGTCGCCCATCACTTCTTCTTCACCAAAATCCCCCACTATCATCTGTTGGAGGCA

ACTCCCGCCATCAAGAAAGCCCTGGAACCTCTGAAAGACACTCAGTACGGATACAAACGG

GAAGTCAACTACAACTGGTTCTTCAAATATCTGCACTACAACGTGACCCTCGACTACTTG

ACCCACAAAGCAAAGGGTGTGCTGCAGTACCGCAGTGGCGTTGAGGCTGCAAAGGCTAAG

AAGGCCCAGTGAACTACAAAATCTCCTGACACGTGTTCATTTTTTTGATTGCCATTTTAT

GTTATAACCAATTTTGAATTTGTTTTTGAAAATTAATTCTCACATATTTCAATGAAAATT

TATGTGCTACTTTTG

**Figure S1.** Optimized fat-2 sequence.
