# Supplementary figures and images for: Transgenic Mice Convert Carbohydrates to Essential Fatty Acids
Source: PLoS One. 2014 May 16;9(5):e97637. doi: 10.1371/journal.pone.0097637 (PMC4023978; doi:10.1371/journal.pone.0097637)

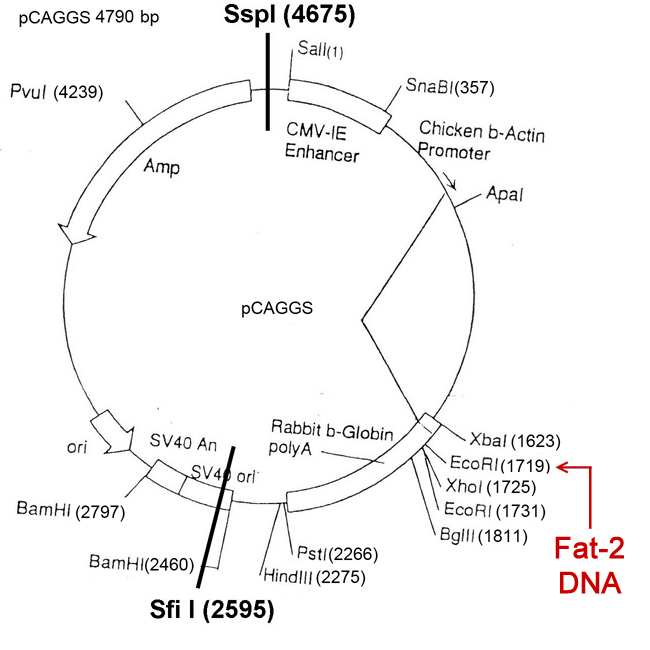


**Figure S2.** The pCAGGS plasmid and fat-2 construct for microinjection.

Supplement: Figure S2 — The pCAGGS plasmid and fat-2 construct for microinjection. (DOC) [file pone.0097637.s002.doc]
