# Supplementary material for: Transgenic Mice Convert Carbohydrates to Essential Fatty Acids
Source: PLoS One. 2014 May 16;9(5):e97637. doi: 10.1371/journal.pone.0097637 (PMC4023978; doi:10.1371/journal.pone.0097637)
Supplement: Table S1 — Composition of the low-PUFA diet and the no-fat diet. (DOC) [file pone.0097637.s003.doc]

**Table S1. Compositions of the low-PUFA diet and the no-fat diet.**

|  | **Low-PUFA diet** | **No-fat diet** |
| --- | --- | --- |
| Protein (kcal%) | 16.8 | 18.6 |
| Carbohydrates (kcal%) | 51.4 | 81.4 |
| Fat (kcal%) | 31.8 | 0.0 |
| **Protein (g)** | **18.4** | **16.8** |
| Casein | 18.1 | 16.5 |
| *DL-Methionine* | 0.3 | 0.3 |
| **Carbohydrates (g)** | **56.4** | **73.5** |
| Corn starch | 24.3 | 31.6 |
| Sucrose | 32.1 | 41.9 |
| **Fat (g)** | **15.5** | **0** |
| Coconut oil | 5.2 | 0 |
| Beef tallow | 10.3 | 0 |
| *Cellulose* | 5 | 5 |
| *Mineral Mix S10001* | 3.5 | 3.5 |
| *Vitamine Mix V10001* | 1 | 1 |
| *Choline Bitartrate* | 0.2 | 0.2 |
| **Total** | **100** | **100** |
| **kcal / g** | 4.39 | 3.61 |

PUFA: polyunsaturated fatty acids.
