# Supplementary material for: Transgenic Mice Convert Carbohydrates to Essential Fatty Acids
Source: PLoS One. 2014 May 16;9(5):e97637. doi: 10.1371/journal.pone.0097637 (PMC4023978; doi:10.1371/journal.pone.0097637)
Supplement: Table S2 — Comparison of the muscle fatty acid profile of mice fed with a low-PUFA diet among the four genotypes. (DOC) [file pone.0097637.s004.doc]

**Table S2.** **Comparison of the muscle fatty acid profile of mice fed with a low-PUFA diet among the four genotypes.**

| **% of FA** | **WT** | **Fat-1** | **Fat-2** | **Omega** |
| --- | --- | --- | --- | --- |
| C12:0 | 3.90±0.22＃ | 3.56±0.69 | 2.61±0.61＃ | 2.97±0.40 |
| C16:0 | 21.56±3.07 | 24.35±1.00 | 24.08±0.47 | 23.58±1.06 |
| C16:1 | 10.71±1.62＃＃△ | 10.76±0.4▲▲* | 7.57±0.61＃＃▲▲ | 8.41±0.80△* |
| C18:0 | 8.69±2.21＃△ | 9.41±1.47 | 12.03±1.36＃ | 12.23±0.68△ |
| C18:1n-9 | 30.75±1.27＃＃△△ | 30.94±1.28▲▲** | 21.37±1.63＃＃▲▲ | 23.51±2.6△△** |
| C18:2n-6(LA) | 7.81±3.17 | 5.72±0.79▲* | 9.13±0.43▲ | 9.68±1.36* |
| C18:3n-3(ALA) |  | 0.23±0.11 |  | 0.46±0.19 |
| C20:4n-6(AA) | 5.28±0.24＃＃△△※※ | 0.46±0.04※※▲▲ | 12.21±0.21＃＃★★▲▲ | 0.69±0.26△△★★ |
| C20:5n-3(EPA) |  | 0.62±0.17 |  | 0.95±0.12 |
| C22:5n-3(DPA) | 0.50±0.25△△ | 1.34±0.37* | 0.67±0.07★★ | 2.11±0.55△△★★* |
| C22:6n-3(DHA) | 3.48±0.38△△※※ | 7.88±0.64※※▲* | 4.05±0.37★★▲ | 11.16±2.15△△★★* |
| SFA | 38.96±0.98 | 40.51±0.35 | 41.15±2.02 | 41.08±0.47 |
| MUFA | 42.87±1.99＃＃△△ | 42.28±1.81▲▲** | 29.71±2.13＃＃▲▲ | 33.03±2.12△△** |
| Total PUFA | 18.19±1.29＃＃△△ | 16.89±1.55▲▲** | 29.01±0.99＃＃★▲▲ | 25.90±1.95△△★** |
| n-6 PUFA | 14.23±1.59＃＃※※ | 6.23±1.16※※▲▲** | 24.29±0.93＃＃★★▲▲ | 11.23±2.43★★** |
| n-3 PUFA | 3.96±0.48△△※※ | 10.66±0.76※※▲▲** | 4.72±0.4★★▲▲ | 14.67±2.09△△★★** |
| n-6/n-3 | 3.60±0.95△△※※ | 0.59±0.10※※▲▲ | 5.18±0.52★★▲▲ | 0.78±0.24△△★★ |

The four genotypes of mice were fed the same low-PUFA diet for about two months and muscle tissue was subject to lipid analysis by gas chromatography. WT: Wild-type; SFA: saturated fatty acids; MUFA: monounsaturated fatty acids; PUFA: polyunsaturated fatty acids; n-6: omega-6; n-3: omega-3; n=3 for each group; ※(WT vs Fat-1), ＃(WT vs Fat-2) , △(WT vs Omega) , ▲(Fat-1 vs Fat-2) , * (Fat-1 vs Omega) , ★(Fat-2 vs Omega), One symbol = P<0.05, Two symbols = P<0.01.
