# Supplementary material for: Transgenic Mice Convert Carbohydrates to Essential Fatty Acids
Source: PLoS One. 2014 May 16;9(5):e97637. doi: 10.1371/journal.pone.0097637 (PMC4023978; doi:10.1371/journal.pone.0097637)
Supplement: Table S3 — Comparison of the liver fatty acid profile of mice fed with a low-PUFA diet among the four genotypes. (DOC) [file pone.0097637.s005.doc]

**Table S3.** **Comparison of the liver fatty acid profile of mice fed with a low-PUFA diet among the four genotypes.**

| **% of FA** | **WT** | **Fat-1** | **Fat-2** | **Omega** |
| --- | --- | --- | --- | --- |
| C12:0 | 1.19±0.32 | 0.93±0.16 | 0.90±0.19 | 0.95±0.21 |
| C16:0 | 27.42±0.65＃ | 26.26±1.35 | 25.18±1.37＃ | 27.01±0.90 |
| C16:1 | 3.82±0.11△※※ | 7.27±0.57※※▲▲* | 4.82±0.58▲▲ | 5.53±1.20△* |
| C18:0 | 7.32±0.83＃ | 9.50±0.41 | 10.13±1.55＃ | 8.84±1.56 |
| C18:1n-9 | 43.36±0.64＃＃△△ | 39.83±0.84▲▲** | 27.79±2.46＃＃▲▲ | 27.37±2.94△△** |
| C18:2n-6(LA) | 2.88±0.62＃＃△△ | 2.31±0.36▲▲** | 7.23±1.06＃＃▲▲ | 8.61±0.99△△** |
| C18:3n-3(ALA) |  | 0.61±0.09 |  | 0.28±0.02 |
| C20:4n-6(AA) | 6.36±0.88＃＃※※ | 3.24±0.87※※▲▲* | 13.64±1.35＃＃★★▲▲ | 5.99±1.16★★* |
| C20:5n-3(EPA) |  | 0.79±0.14 |  | 1.14±0.16 |
| C22:5n-3(DPA) |  | 0.61±0.09 |  | 0.37±0.06 |
| C22:6n-3(DHA) | 2.57±0.07＃△△※※ | 3.86±0.13※※** | 3.75±0.25＃★★ | 8.92±0.82△△★★** |
| SFA | 36.70±0.83 | 37.49±1.27 | 37.33±2.25 | 37.90±2.09 |
| MUFA | 48.78±1.00＃＃△△ | 49.38±0.72▲▲** | 33.87±3.05＃＃▲▲ | 34.21±3.75△△** |
| Total PUFA | 14.44±1.32＃＃△△ | 13.16±0.77▲▲** | 28.82±1.55＃＃▲▲ | 27.90±2.50△△** |
| n-6 PUFA | 11.86±1.33＃＃△△※※ | 7.28±0.58※※▲▲** | 25.03±1.69＃＃★★▲▲ | 17.38±1.76△△★★** |
| n-3 PUFA | 2.57±0.07＃△△※※ | 5.88±0.40※※▲▲** | 3.75±0.25＃★★▲▲ | 10.52±0.74△△★★** |
| n-6/n-3 | 4.62±0.55＃＃△△※※ | 1.24±0.11※※ ▲▲ | 6.65±0.82＃＃★★▲▲ | 1.65±0.05△△★★ |

The four genotypes of mice were fed the same low-PUFA diet for about two months and liver tissue was subject to lipid analysis by gas chromatography. WT: Wild-type; SFA: saturated fatty acids; MUFA: monounsaturated fatty acids; PUFA: polyunsaturated fatty acids; n-6: omega-6; n-3: omega-3; n=3 for each group; ※(WT vs Fat-1), ＃(WT vs Fat-2) , △(WT vs Omega) , ▲(Fat-1 vs Fat-2) , * (Fat-1 vs Omega) , ★(Fat-2 vs Omega), One symbol = P<0.05, Two symbols = P<0.01.
