# Supplementary material for: Transgenic Mice Convert Carbohydrates to Essential Fatty Acids
Source: PLoS One. 2014 May 16;9(5):e97637. doi: 10.1371/journal.pone.0097637 (PMC4023978; doi:10.1371/journal.pone.0097637)
Supplement: Table S4 — Comparison of the tail fatty acid profile of mice fed with a low-PUFA diet among the four genotypes. (DOC) [file pone.0097637.s006.doc]

**Table S4.** **Comparison of the tail fatty acid profile of mice fed with a low-PUFA diet among the four genotypes.**

| **% of FA** | **WT** | **Fat-1** | **Fat-2** | **Omega** |
| --- | --- | --- | --- | --- |
| C12:0 | 2.71±0.23＃＃ | 2.64±0.09▲ | 2.17±0.25＃＃▲ | 2.46±0.16 |
| C16:0 | 14.41±0.89＃＃△ | 14.92±1.09▲* | 18.04±1.11＃＃▲ | 17.54±1.47△* |
| C16:1 | 15.57±1.08＃ | 15.56±0.94▲ | 10.48±3.55＃▲ | 12.07±0.83 |
| C18:0 | 5.45±0.89＃＃△ | 6.51±0.37▲ | 10.34±2.73＃＃▲ | 9.02±1.08△ |
| C18:1n-9 | 44.03±2.3＃＃△△ | 47.14±1.41▲▲** | 34.57±2.25＃＃▲▲ | 34.21±1.47△△** |
| C18:2n-6(LA) | 2.62±0.16＃＃△△ | 1.96±0.14▲▲** | 9.74±1.83＃＃▲▲ | 8.64±0.97△△** |
| C18:3n-3(ALA) |  | 1.78±0.26 |  | 3.74±0.37 |
| C20:4n-6(AA) | 3.02±0.45 |  | 5.53±0.50 |  |
| C20:5n-3(EPA) |  | 1.05±0.16 |  | 1.19±0.11 |
| C22:5n-3(DPA) |  | 1.03±0.25 |  | 1.15±0.16 |
| C22:6n-3(DHA) | 0.93±0.23△△※※ | 1.69±0.33※※ | 1.35±0.33★ | 1.96±0.16△△★ |
| SFA | 29.41±1.55 | 26.99±1▲* | 33.16±3.42▲ | 32.79±2.73* |
| MUFA | 62.39±2.19＃＃△△ | 64.96±1.38▲▲** | 47.65±3.55＃＃▲▲ | 49.02±2.15△△** |
| Total PUFA | 8.20±0.63＃＃△△ | 8.05±0.38▲▲** | 19.20±1.57＃＃▲▲ | 18.20±1.18△△** |
| n-6 PUFA | 7.27±0.41＃＃△※※ | 2.49±0.32※※▲▲** | 17.85±1.5＃＃★★▲▲ | 10.16±1.62△★★** |
| n-3 PUFA | 0.93±0.23△△※※ | 5.55±0.7※※▲▲** | 1.35±0.33★★▲▲ | 8.04±0.44△△★★** |
| n-6/n-3 | 8.07±1.38＃＃△△※※ | 0.46±0.11※※▲▲ | 13.81±3.87＃＃★★▲▲ | 1.27±0.27△△★★ |

The four genotypes of mice were fed the same low-PUFA diet for about two months and tail tissue was subject to lipid analysis by gas chromatography. WT: Wild-type; SFA: saturated fatty acids; MUFA: monounsaturated fatty acids; PUFA: polyunsaturated fatty acids; n-6: omega-6; n-3: omega-3; n=3 for each group; ※(WT vs Fat-1), ＃(WT vs Fat-2) , △(WT vs Omega) , ▲(Fat-1 vs Fat-2) , * (Fat-1 vs Omega) , ★(Fat-2 vs Omega), One symbol = P<0.05, Two symbols = P<0.01.
