# Supplementary material for: Transgenic Mice Convert Carbohydrates to Essential Fatty Acids
Source: PLoS One. 2014 May 16;9(5):e97637. doi: 10.1371/journal.pone.0097637 (PMC4023978; doi:10.1371/journal.pone.0097637)
Supplement: Table S5 — Comparison of the tail fatty acid profile of mice fed with a non-fat diet among the four genotypes. (DOC) [file pone.0097637.s007.doc]

**Table S5.** **Comparison of the tail fatty acid profile of mice fed with a non-fat diet among the four genotypes.**

| **% of FA** | **WT** | **Fat-1** | **Fat-2** | **Omega** |
| --- | --- | --- | --- | --- |
| C12:0 | 2.75±0.24※ | 2.12±0.34※* | 2.35±0.08 | 2.70±0.34* |
| C16:0 | 15.53±4.05 | 15.49±0.36 | 15.35±0.60 | 14.97±0.93 |
| C16:1 | 16.91±2.46※ | 13.35±0.62※ | 15.71±1.22 | 15.20±1.23 |
| C18:0 | 7.29±4.52 | 7.67±0.77 | 6.69±1.01 | 8.54±2.76 |
| C18:1n-9 | 45.19±5.25＃ | 48.88±0.60▲▲** | 38.93±2.12＃▲▲ | 39.78±1.35** |
| C18:2n-6(LA) | 3.11±0.59＃＃△△ | 2.32±0.38▲▲** | 9.05±1.05＃＃★▲▲ | 7.75±0.45△△★** |
| C18:3n-3(ALA) |  | 1.11±0.43 |  | 1.98±0.12 |
| C20:4n-6(AA) | 2.73±0.26 |  | 4.33±0.33 |  |
| C20:5n-3(EPA) |  | 0.53±0.10 |  | 0.89±0.5 |
| C22:5n-3(DPA) |  | 0.55±0.11 |  | 0.70±0.04 |
| C22:6n-3(DHA) | 0.66±0.14△※※ | 1.81±0.70※※▲▲ | 0.65±0.09★▲▲ | 1.58±0.21△★ |
| SFA | 28.85±7.64 | 28.24±0.33 | 27.78±1.56 | 29.06±3.43 |
| MUFA | 64.09±8.13 | 65.46±1.05▲* | 57.14±2.01▲ | 56.98±2.65* |
| Total PUFA | 7.06±0.89＃＃△△ | 6.32±0.71▲▲** | 15.10±0.65＃＃▲▲ | 13.97±0.78△△** |
| n-6 PUFA | 6.40±0.75※※＃＃△△ | 2.32±0.38※※▲▲** | 14.44±0.74＃＃★★▲▲ | 8.82±0.75△△★★** |
| n-3 PUFA | 0.66±0.14△△※※ | 4.00±0.66※※▲▲** | 0.65±0.09★★▲▲ | 5.16±0.28△△★★** |
| n-6/n-3 | 9.93±1.20＃＃△△※※ | 0.59±0.16※※▲▲ | 22.44±3.82＃＃★★▲▲ | 1.71±0.18△△★★ |

The four genotypes of mice were fed the same non-fat diet for about two months and tail tissue was subject to lipid analysis by gas chromatography. WT: Wild-type; SFA: saturated fatty acids; MUFA: monounsaturated fatty acids; PUFA: polyunsaturated fatty acids; n-6: omega-6; n-3: omega-3; n=3 for each group; ※(WT vs Fat-1), ＃(WT vs Fat-2) , △(WT vs Omega) , ▲(Fat-1 vs Fat-2) , * (Fat-1 vs Omega) , ★(Fat-2 vs Omega), One symbol = P<0.05, Two symbols = P<0.01.
